# Identification and characterisation of common glow-worm RNA viruses

**DOI:** 10.1007/s11262-019-01724-5

**Published:** 2020-01-03

**Authors:** Lumi Viljakainen, Anna-Maria Borshagovski, Sami Saarenpää, Arja Kaitala, Jaana Jurvansuu

**Affiliations:** Department of Ecology and Genetics, University of Oulu, Pentti Kaiteran katu 1, 90014 Oulu, Finland

**Keywords:** Common glow-worm, RNA viruses, Rna-sequencing, Virus transmission

## Abstract

The common glow-worms (*Lampyris noctiluca*) are best known for emission of green light by their larvae and sexually active adult females. However, both their DNA and RNA viruses remain unknown. Glow-worms are virologically interesting, as they are non-social and do not feed as adults, and hence their viral transmission may be limited. We identified viral sequences from 11 different virus taxa by the RNA-sequencing of two Finnish populations of adult glow-worms. The viruses represent nine different virus families and have negative, positive, or double-stranded RNA genomes. We also found a complete retroviral genome. Similar viral sequences were found from the sequencing data of common eastern firefly of North America, a species belonging to the same family (Lampyridae) as that of the common glow-worm. On average, an individual glow-worm had seven different RNA virus types and most of them appeared to establish a stable infection since they were found from glow-worms during two consecutive years. Here we present the characterization of load, prevalence, and interactions for each virus. Most of the glow-worm RNA viruses seem to be transmitted vertically, which may reflect the biology of glow-worms as non-social capital breeders, i.e., they invest stored resources in reproduction.

**Electronic supplementary material:**

The online version of this article (10.1007/s11262-019-01724-5) contains supplementary material, which is available to authorized users.

## Introduction

The common glow-worm (*Lampyris noctiluca*) is a nocturnal beetle, whose larvae and sedentary females emit highly visible green light. Larva glow is a warning signal for distastefulness [1–3] and adult females produce green light to attract flying males [4, 5]. Glow-worms are capital breeders: they feed only as larvae, rendering this stage critical for storing energy, and potentially, for acquiring commensal and pathogenic organisms. Larval development takes one to four years and adult lifespan is of few weeks [4]. Most adult females glow for only one or a few nights to attract males, which is followed by mating, and soon after, they lay their eggs and die. The common glow-worm is widely distributed across the northern hemisphere, from Portugal to China and from Spain to Finland [4, 6].

Glow-worm populations seem to be isolated from each other due to local differences in their emergence time and females’ inability to fly. This lack of conspecific contact makes glow-worms epidemiologically interesting. Larvae are predators feeding mainly on slugs and snails, and at early stages the larvae may forage together [4]. Individuals from different populations have limited contact with each other, and likewise, individuals within one population meet perhaps only at birth, during larval stage, and mating [4, 7]. Thus, horizontal virus transmission, that is, transmission between individuals of the same generation, is presumably quite limited. So far the only viruses found in Lampyridae family, the two orthomyxo-like viruses found in the North American common eastern firefly (*Photinus pyralis*), were observed to be transmitted vertically, that is, from parent to offspring [8].

We identified viral sequences from two Finnish glow-worm populations and studied their presence in different life stages and tissues in Finnish and English glow-worms. We identified 11 RNA viruses from adult glow-worms, four positive-stranded, three negative-stranded, three double-stranded, and one retrovirus. Most of the viruses formed stable infections as they were obtained from glow-worms collected in two different years. The glow-worm viruses are found in egg tissue as well as unfed larvae, which suggests that most of them may be transmitted vertically from parents to offspring.

## Material and methods

### Glow-worms

The data consists of several cohorts: (1) Adult female and male glow-worm samples used for RNA-sequencing were collected from two Finnish populations: Nine females and four males from Konnevesi (in the middle of Finland, N62° 37′, E26° 20′, collected in July and August 2017); and eight females and eight males from Hanko (southern Finland, N59° 53′, E23° 06′, collected in June and July 2017). (2) Adult female samples, used for qPCR analyses, were collected in June and July, 2018, from two populations, four females from Äänekoski (in the middle of Finland, N62° 36′, E25° 43′), and five females from Tvärminne Zoological Station (southern Finland, N59° 50′, E23°14′). (3) Laboratory-reared larvae from different mothers were used for qPCR analyses. Mothers of three of these larvae were collected from southern Finland (Lohja, Öby, and Särkisalo) and mated in 2016, and three larvae with unknown relations were collected from England (Princes Risborough, N51° 43′, W0° 50′). These six larvae were reared and kept in the laboratory for two years and fed with organic cat food and terrestrial and aquatic snails collected from southern Finland. (4) Two-week-old larval offspring of two parental glow-worms (collected and mated in June 2018) were obtained from Tvärminne Zoological Station. The larvae (*N* = 12) hatched one month after egg-laying and were kept alive, unfed for 2 weeks. Online Resource 1 shows a map of sample collection sites. All the insects were stored in RNAlater until RNA extraction.

### RNA isolation, RNA-seq, and cDNA

Female lanterns and male heads of samples from 2017 were individually dissected and used for RNA extraction, using RNeasy Micro Kit (Qiagen). The RNA samples were sent to BGI Tech Solutions for poly-A selected and strand-specific library preparation using Illumina TruSeq Stranded mRNA Library Prep Kit and RS-122–2101 kit. The libraries were sequenced with Illumina HiSeq4000 producing 100 bp paired-end reads. Samples from 2018 were isolated with Trizol (ThermoFisher). cDNA was prepared using 500 ng total-RNA with ReverAid H Minus First Strand cDNA synthesis kit (ThermoFisher), as per the manufacturer’s protocol.

### Bioinformatics analysis

The raw RNA-seq reads from the 2017 samples were trimmed for adapters and low-quality nucleotides (Q < 20) and filtered for reads less than 36 nt in length. The trimmed reads were pooled and assembled using Trinity v2.3.2 with in silico normalization [9]. Contigs over 1000 nt were aligned to each other using CAP3 [10]. Contigs were pooled and searched using Blastx v2.3.0 [11] (an *e*-value < 10^–10^ and culling limit 1) against invertebrate virus protein sequences using NCBI virus protein RefSeq (downloaded in May, 2018). Contigs from individual data were translated to protein sequences with Transdecoder (github.com/TransDecoder), and only the longest of protein coding sequence over 160 aa was selected for search with Blastp against the invertebrate virus proteins. Virus positive contigs were pooled and aligned with each other with CAP3 and searched using Blastx, as above. Virus contigs were manually checked for complete or nearly complete viral genomes using NCBI ORFfinder (www.ncbi.nlm.nih.gov/orffinder/). All the identified viral sequences were seeded into NOVOplasty [12] with the original RNA-sequencing data to make sure that the viral sequences were as complete as possible. The data are deposited into at NCBI (PRJNA577041). Coverage and alignment of the reads to the virus sequences were studied with IGV [13] so that at least two reads were covering the reference sequence at any site. Remapping of reads from each glow-worm separately to virus sequences was done with BWA [14].

NCBI Blastp (nr) was used to identify possible domains and identical protein sequences: identity describes, which percent of characters in the query sequence is identical with the target sequence and coverage describes how much of the query sequence is covered by the target sequence. HHpred [15] was used to search for remote homologous proteins based on protein structure prediction. RNAfold (rna.tbi.univie.ac.at/cgi-bin/RNAWebSuite/RNAfold.cgi) was used to predict stable genomic RNA structures.

Similar virus sequences to glow-worm viruses were searched with Blastx from other insects (taxid:6960) from NCBI’s transcriptome shotgun assembly sequence database (TSA). We set Blastx bit score, which takes into account the alignment length, mismatches and gaps, above 200 for positive hits.

## Phylogenies

Phylogenies were reconstructed from the RdRP amino acid sequences of each virus and 19 most similar hits in Blastp, RSA TSA hits (Table 2), and virus sequences from Shi et al. [16]. Sequences were aligned using E-INS-I method in MAFFT v7.313 [17] and prior to phylogenetic analysis the alignments were trimmed using trimAl v1.2 [18]. Amino acid substitution model was selected using ProtTest 3 [19] and phylogeny reconstruction was performed using PhyML v.3.0 [20]. Phylogenies were processed with iTOL [21].

## Quantitative-PCR (qPCR)

qPCR was performed with virus-specific primers (Online Resource 2) using EvaGreen (Solis BioDyne) kit, as per manufacturers’ protocol. Standards for qPCR were created with virus-specific primers using Phusion enzyme (ThermoFisher). The PCR products were isolated with GeneJET gel extraction kit (ThermoFisher). Only the results with over 10 viral sequences were analyzed further.

### Statistical analysis

All statistical analyses were performed in R v3.5.0 (www.R-project.orf/). Pairwise analysis of virus reads was done by Spearman’s correlation using multiple testing p-value correction.

## Results

### Virus identification

**Fig. 1:**
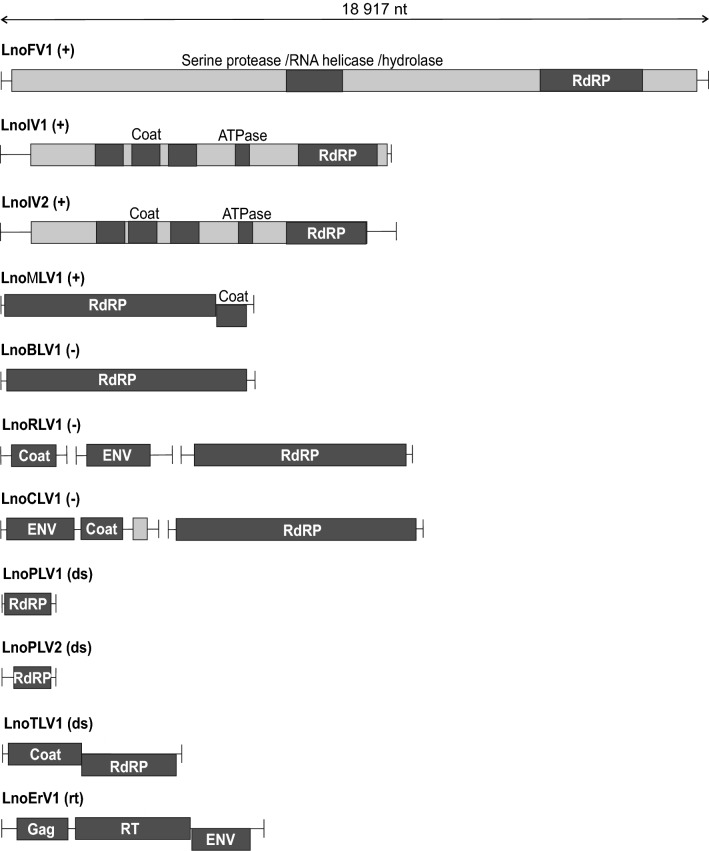
Genome organization of the glow-worm viruses: The virus genome type is indicated in parenthesis, after virus name: positive ( +), negative (−), double-stranded (ds), or retrovirus (rt). Line denotes virus sequence and boxes open reading frames (ORFs). Dark gray boxes represent domains identified by HHpred or Blastp. The size in nucleotides is indicated for the largest virus and the sizes of the other viruses are in scale to that. LnoMLV1 has two ORFs in different reading frames presented by the two ORF boxes aligned above and below the sequence line. *RdRP* RNA-dependent RNA polymerase, *Gag* capsid, *ENV* envelope, and *RT* reverse transcriptase

We identified 11 RNA viruses by whole-transcriptome sequencing of 29 adult glow-worms. The samples were from two Finnish populations collected in 2017, four males (heads) and eight females (lanterns) from Konnevesi Research Station and eight males (heads) and nine females (lanterns) from Hanko (Online Resource 1). The tissues were chosen because they are important in sexual signaling. Viral sequences were assembled from pooled sample with the Trinity assembler (Table 1) and virus contigs were identified by protein similarity search against National Center for Biotechnology Information (NCBI) virus protein database. The virus sequences were deposited into NCBI GenBank database (Table 2). The viruses were named according to their genomic organization (Fig. 1) and phylogenetic relationship to known insect viruses (Online Resources 5–12): *Lampyris noctiluca* flavivirus 1, *Lampyris noctiluca* iflavirus 1, *Lampyris noctiluca* iflavirus 2, *Lampyris noctiluca* macula-like virus 1, *Lampyris noctiluca* bunya-like virus 1, *Lampyris noctiluca* rhabdo-like virus 1, *Lampyris noctiluca* chuvirus-like virus 1, *Lampyris noctiluca* partiti-like virus 1, *Lampyris noctiluca* partiti-like virus 2, *Lampyris noctiluca* totivirus-like virus 1, and *Lampyris noctiluca* errantivirus 1. The sequence data were strand-specific and allowed us to separate the negative-strand replicative intermediates of positive-strand viruses and positive-strand transcripts of negative-strand viruses (Online Resource 3). The strand-specific data indicated that all identified positive-strand RNA viruses were replicating in the glow-worms. However, as the production of the sequencing library involved poly-A enrichment that biases the ratios of the virus transcripts, the strand-specific data were not analyzed further.

**Table 1:** Virus sequence identification from the pooled glow-worm RNA-sequencing data

Virus	Full virus name	Family	Genome type	Length (nt)	Reads	Coverage^a^	RPKM^b^
LnoFV1	*Lampyris noctiluca* flavivirus 1	*Flaviviridae*	ssRNA +	18,912	40,858	216.04	1.16
LnoIV1	*Lampyris noctiluca* iflavirus 1	*Iflaviridae*	ssRNA +	10,286	383,062	3724.11	19.91
LnoIV2	Lampyris noctiluca iflavirus 2	*Iflaviridae*	ssRNA +	10,369	1,316,524	12,696.73	67.9
LnoMLV1	*Lampyris noctiluca* macula-like virus 1	*Tymoviridae*	ssRNA +	6733	36,646,669	544,284.41	2910.35
LnoBLV1	*Lampyris noctiluca* bunya-like virus 1	*Bunyaviridae*	ssRNA -	6774	1447	21.36	0.11
LnoRLV1	*Lampyris noctiluca* rhabdo-like virus 1	*Rhabdoviridae*	ssRNA -	6183	1003	16.22	0.09
				2571	3408	132.56	0.71
				1743	1794	102.93	0.55
LnoCLV1	*Lampyris noctiluca* chuvirus-like virus 1	*Chuviridae*	ssRNA -	6829	3727	54.58	0.29
				4489	27,337	608.98	3.26
LnoPLV1	*Lampyris noctiluca* partiti-like virus 1	*Partitiviridae*	dsRNA	1462	71	4.86	0.03
LnoPLV2	*Lampyris noctiluca* partiti-like virus 2	*Partitiviridae*	dsRNA	1461	11,206	767.01	4.1
LnoTLV1	*Lampyris noctiluca* totivirus-like virus 1	*Totiviridae*	dsRNA	4761	468	9.83	0.05
LnoErV1	*Lampyris noctiluca* errantivirus 1	*Metaviridae*	Retrovirus	6931	1566	22.6	0.00012

^a^All virus-specific bases divided by virus length

^b^Reads Per Kilobase of transcript per Million mapped reads

**Table 2:** Glow-worm viruses identified in this study and existence of similar viruses in other insects

Virus	GenBank ID	Insect with similar SRA sequences (TSA blastx score > 200)	Common name (description)
LnoFV1	MH620810	No	
LnoIV1	MH620811	*Ceratitis capitata* (JAB85953.1)	The mediterranean fruit fly
		*Cuerna arida* (JAS52200.1)	(sharpshooter)
		Glossina morsitans morsitans (ACY69873.1)	Tsetse fly
		*Lygus hesperus* (JAG64378.1)	The western tarnished plant bug
		*Photinus pyralis* (JAV83398.1)	The eastern firefly
LnoIV2	MH620812	*Bactrocera dorsalis* (JAC58554.1)	The oriental fruit fly
		*Bactrocera latifrons* (JAI25725.1)	Solanum fruit fly
		*Ceratitis capitata* (JAC04636.1)	
		*Cuerna arida* (JAS44129.1)	
		*Fopius arisanus* (JAG76255.1)	(Braconid wasp)
		*Glossina morsitans morsitans* (ADD18747.1)	
		*Lygus hesperus* (JAQ02432.1)	
		*Panstrongylus lignarius* (JAW07564.1)	(kissing bug)
		*Pectinophora gossypiella* (JAT85648.1)	Pink bollworm (moth)
		*Photinus pyralis* (JAV89834.1)	
		*Zeugodacus cucurbitae* (JAD00225.1)	The melon fly
LnoMLV1	MH620813	no	
LnoBLV1	MH620814	*Homalodisca liturata* (JAS79980.1)	Smoketree sharpshooter
		*Lygus hesperus* (JAG07595.1)	
		*Pararge aegeria* (JAA88706.1)	The speckled wood butterfly
		*Photinus pyralis* (JAV88611.1)	
LnoRLV1	MH620815	*Ceratitis capitata* (JAB89047.1)	
	MH620816	*Clastoptera arizonana* (JAS12225.1)	The Arizona spittlebug
	MH620817	*Corethrella appendiculata* (JAB55768.1)	(midge)
		*Cuerna arida* (JAS60519.1)	
LnoCLV1	MH620818	*Aedes albopictus* (JAV46700.1)	Asian tiger mosquito
	MH620819	*Anopheles darlingi* (MBW69371.1)	Mosquito (malaria)
		*Anopheles triannulatus* (MBW43032.1)	Mosquito
		*Schizaphis graminum* (MBY23430.1)	Wheat aphid
		*Sipha flava* (MBY84526.1)	Yellow sugarcane aphid
LnoPLV1	MH620820	*Fopius arisanus* (JAG72135.1)	
		*Graphocephala atropunctata* (JAT20972.1)	Blue-green sharpshooter
		*Nyssomyia neivai* (JAV02130.1)	The sand fly
		*Photinus pyralis* (JAV58376.1)	
LnoPLV2	MH620821	No	
LnoTLV1	MH620822	No	
LnoErV1	MH620823	*Aedes albopictus* (JAV46740.1)	
		*Anoplophora glabripennis* (JAB63837.1)	Asian long-horned beetle
		*Clastoptera arizonana* (JAS19451.1)	
		*Lygus hesperus* (JAG18612.1)	
		*Panstrongylus lignarius* (JAW07754.1)	
		*Photinus pyralis* (JAV62639.1)	
		*Triatoma infestans* (JAC14661.1)	(Kissing bug)

*RSA* NBCI’s Sequence Read Archive, *TSA* NCBI’s transcriptome shotgun assembly sequence database, in green are shown virus RdRP sequences that are present in virus phylogenies

We analyzed also virus sequence variation between the samples (Online Resource 3). However, only fraction of the glow-worms had complete virus sequences without gaps and thus the analysis produced only descriptive information. When considering only southern population females that had at least two complete virus sequences per group, highest frequency of variable sites were with iflaviruses (LnoIV1: 0.0097 variable sites/genome size/sample number and LnoIV2: 0.0152), and lowest with flavivirus LnoFV1 (0.00097) and partitivirus LnoPV1 (0.0010). Frequency of variable sites for partitivirus LnoPLV2 was 0.0016 variable sites/genome size/sample number.

### *Lampyris noctiluca* flavivirus 1

Flaviviruses are positive-strand RNA viruses and include notorious arboviruses with mosquito (Dengue, Zika, Yellow Fever, West Nile, Japanese encephalitis) and tick vectors (tick-borne encephalitis virus). *L. noctiluca* flavivirus 1 (LnoFV1) is 18,917 nt long and encodes a 6152 aa polyprotein. Blastp search results indicated protein sequence similarity to the putative polyprotein of Drosophila flavivirus, Takaungu virus (19% coverage with 59% identity, NCBI taxonomy ID = 1,807,800). HHpred found sites in the LnoFV1 sequence similar to Serine protease NS3/RNA helicase/hydrolase of classical swine fever virus (*e*-value = 3.6e-25, PDB ID = 5WX1_A) and methyltransferase and RNA-dependent RNA polymerase (RdRP) of Japanese encephalitis virus (*e*-value 2e-34, protein data base id: 4K6M_A). Both swine fever virus and Japanese encephalitis virus are flaviviruses. On the basis of the phylogenetic analysis, LnoFV1 was most similar to Drosophila flavi-like viruses, Takaungu virus and Hermitage virus (1,807,799) (Online Resource 5) [22]. RNAfold (rna.tbi.univie.ac.at) was used to predict secondary structures at the 5′ and 3′ untranslated regions and LnoFV1 was found to have highly structured 3′ UTR typical for flaviviruses [23].

### *Lampyris noctiluca* iflavirus 1 and 2

Iflaviruses are insect-infecting positive-strand RNA viruses coding for a single polyprotein. *Lampyris noctiluca* iflavirus 1 (LnoIV1) is 10,339 nt long and codes for a polyprotein of 3134 aa. Blastp indicates polyprotein similarity (47% coverage and 28% identity) to a hypothetical protein of Hubei picorna-like virus 31 (1,923,111) from odonata mix [16]. According to HHpred, the polyprotein had sites similar to human rhinovirus 16 coat protein (*e*-value = 1.6e-23, 1AYM_2), coat protein of iflavirus Deformed wing virus (*e*-value = 2e-42, 5MV5_C), capsid protein of iflavirus Slow bee paralysis virus (*e*-value = 1.9e-17, 5J98_A), ATPase of enterovirus 2C (*e*-value = 4.6e-13 5GQ1_C), and RdRP of human polio virus 1 (*e*-value = 9.9e-65, 2IJD_1). All the HHpred identified viruses belonged to the *Picornavirales* order. Based on the phylogenetic analysis LnoIV1 was most closely related to unclassified bat Rolda virus (1,911,103) (Online Resource 6). Stable RNA structures, which could function as an internal ribosomal entry site (IRES), were identified by RNAfold on the first 729 nucleotides.

*Lampyris noctiluca* iflavirus 2 (LnoIV2) is 10,428 nt long and codes a polyprotein of 3132 aa. Blastp search shows polyprotein similarity (73% coverage and 26% identity) to unclassified bat virus polyprotein of Rolda virus (1,911,103). According to HHpred, the polyprotein had sites similar to the capsid proteins of Slow bee paralysis virus (*e*-value = 1.9E-26, 5J98_B and *e*-value = 1.7e-47 5J98_C) and Deformed wing virus (*e*-value = 1.6E-21, 5MV5_A), ATPase of enterovirus 2C (*e*-value = 1.7E-10, 5GQ1_B), and RdRP of human poliovirus 1 (*e*-value = 1.2e-62, 22IJD_2). All HHpred identified viruses belong to the *Picornavirales* order. According to phylogenetic analysis, LnoIV2 was most similar to Hubei picorna-like virus 31 (1,923,111) from odonata mix [16] (Online Resource 6). Stable RNA structures, which could function as IRES, were identified on the first 1002 nucleotide. Polyprotein similarity between *L. noctiluca* iflavirus 1 and 2 is 29.5% with 33% coverage. Thus, the sequence identity at the amino acid level between the capsid proteins is less than 90% and the viruses represent separate new species according to the International Committee on Taxonomy of Viruses (ICTV).

### *Lampyris noctiluca* macula-like virus 1

*Tymoviridae* are positive-strand RNA viruses without an envelope. Tymoviruses infect mainly plants but they have also been found in insects, such as, bees (NCBI txid:1,682,186) [24, 25], silkworms (288,456 and 2,065,033) [26], mites (1,682,187 and 1,005,993) [25], and mosquitoes (1,236,047) [27].

*Lampyris noctiluca* macula-like virus 1 (LnoMLV1) is 6744 nt long with two open reading frames (ORFs). The longer ORF codes for a protein of 1920 aa, which was similar to an RdRP and a hypothetical protein of Bombyx mori Macula-like virus (txid:288,456, 84% coverage with 56% identity) according to Blastp search. The best hit by HHpred was a replicase of Tomato mosaic virus (*e*-value = 3e-24, pdb:3VKW_A), and several shorter hits to viruses of the *Tymoviridae* family. Shorter ORF, coding for a protein of 231 aa, was similar (98% coverage and 45% identity) to a coat protein of Bee Macula-Like virus 2 (2,094,260), according to Blastp search. HHpred showed that the ORF was similar to plant tymovirus desmodium yellow mottle virus capsid protein (*e*-value = 6.8e-59, 1DDL_C). Species demarcation criteria for *Maculavirus* genus is by ICTV less than 90% identity in capsid protein. Based on phylogenetic analysis, LnoMLV1 was most similar to Bee Macula-Like virus 2 (2,094,260) (Online Resource 7).

### *Lampyris noctiluca* bunya-like virus 1

Bunyaviruses have tripartite negative-strand RNA genomes coding for RdRP, nucleoprotein, and envelope proteins. Only the 6774 nt RdRP genome was found for the *Lampyris noctiluca* bunya-like virus 1 **(**LnoBLV1). The 2182 aa long protein was most similar to Hubei insect virus 1 RdRP (1,922,897) and several bat bunyaviruses (98% coverage and 39% identity, AOY18798.1-AOY18800.1) according to Blastp search. HHpred showed that the protein was similar to RNA polymerase L (5AMR_A) of bunyavirus “la Crosse”. Phylogenetic analysis placed LnoBLV1 closest to hypothetical protein of Smoketree sharpshooter (Table 2), Hubei insect virus 1 (1,922,897) from arthropod mix (Online Resource 8) [16].

### *Lampyris noctiluca* rhabdo-like virus 1

Rhabdoviruses are monopartite negative-strand RNA viruses. *L. noctiluca* rhabdo-like virus 1 (LnoRLV1) was found in three parts. The longest transcript of 6218 nt codes for a protein of 1924 aa, which according to Blastp search, is similar to Hubei rhabdo-like virus 3 (1,923,187) RdRP from Coleoptera mix (99% coverage and 34% identity). According to HHpred, the protein was similar to vesicular stomatitis virus L polymerase (*e*-value = 3.8e-190, 5A22_A). The second transcript of 2571 nt encodes a protein of 603 aa. Blastp search indicated the similarity of the protein to Hubei rhabdo-like virus 3 hypothetical protein 1 (80% coverage and 23% identity), and HHpred analysis showed that the protein was somewhat similar to fusion glycoprotein of Hendra virus (*e*-value = 0.00002, 5EJB_C), which suggests that the protein codes for an envelope. The smallest transcript of 1743 nt codes for a protein of 426 aa, which Blastp search found most similar to putative glycoprotein of Hubei rhabdo-like virus 3 (39% coverage and 28% identity), and HHpred showed a weak similarity to p40 nucleoprotein of Borna disease virus (*e*-value = 23, 1N93_X) from the *Mononegavirales* order, to which the rhabdoviruses belong. Thus, the smallest transcript most probably codes for a capsid protein. Based on the phylogenetic analysis, LnoRLV1was most similar to Hubei rhabdo-like virus 3 (1,923,187) isolated from a beetle mix [16] (Online Resource 9).

### *Lampyris noctiluca* chuvirus-like virus 1

*Chuviridae* is a negative-stranded RNA virus family within the order *Mononegavirales,* with variable genome structures i.e. segmented, non-segmented, and circular [28]. LnoCLV1 genome had two segments, the larger genome of 6829 nt and encodes a protein of 2206 aa and the smaller segment that was 4489 nt long, with three ORFs coding for 693, 407, and 115 aa long proteins. According to Blastp search, the protein from the larger genome segment was similar to RdRP of Hubei chuvirus-like virus 3 (100% coverage and 43% identity, 1,922,858), and HHpred showed similarity to vesicular stomatitis virus L polymerase (*e*-value = 5.2e-185, 5A22_A). According to Blastp search, the 693 amino acids protein was similar to putative glycoprotein of Hubei chuvirus-like virus 3 (91% coverage and 49% identity) and HHpred showed similarity to the envelope glycoprotein of human herpesvirus 1 (*e*-value = 1.6e-27), which suggests that the ORF codes for an envelope protein. The 407 aa protein was similar to a hypothetical protein of Hubei chuvirus-like virus 3 (Blastp: 95% coverage and 37% identity), and HHpred showed protein structural similarity to p40 nucleoprotein of Borna disease virus (*e*-value = 2.6, 1N93_X) suggesting that the ORF may code for a coat protein. For the shortest protein of 115 aa, neither Blastp nor HHpred found any significantly similar proteins. According to phylogenetic analysis, LnoCLV1 was most similar to Hubei chuvirus-like virus 3 (1,922,858) (Online Resource 9), which has been isolated from Odonata mix and has a monopartite genome [16].

### *Lampyris noctiluca* partiti-like virus 1 and 2

Partitiviruses are small bipartite double-stranded RNA viruses coding for RdRP and capsid proteins. We could only find the RdRP coding segment for two partitivirus-like viruses identified from the glow-worms. *Lampyris noctiluca* partiti-like virus 1 (LnoPLV1) genome was 1462 nt long and codes for a protein of 377 aa. According to Blastp search, the protein is similar to RdRP of Hubei partiti-like virus 31 (1,923,038, 93% coverage and 72% identity). HHpred showed that the protein sequence was similar to RdRP of negative-strand RNA virus, Thosea asigna virus (*e*-value = 1.5e-36, 5CX6_B). In phylogenetic analysis LnoPLV1 was closest to Hubei partiti-like virus 31 (Online Resource 10) isolated from spider mix. Only the RdRP genome segment was identified in the Hubei partiti-like virus 31 [16].

*Lampyris noctiluca* partiti-like virus 2 (LnoPLV2) genome segment was 1461 nt long and encoded a 436 aa protein. Blastp search showed similarity to RdRP of Hubei partiti-like virus 51 (90% coverage and 42% identity, 1,923,060), which was closest in the phylogenetic analysis also (Online Resource 10). Hubei partiti-like virus 51 was identified from Chinese land snail mix, and only RdRP segment was found, as observed in LnoPLV2, HHpred showed that LnoPLV2 RdRP is most similar to Mengo virus RdRP (8.6e-37, 4NYZ_A). Mengo virus belongs to the *Picornaviridae* family. LnoPLV1 and LnoPLV2 are separate species as their had only 28.6% identity over RdRP residues (57.6% coverage) and were only distantly related by phylogenetic analysis (Online Resource 10).

### *Lampyris noctiluca* totivirus-like virus 1

Totiviruses are double-stranded RNA viruses without an envelope. *L. noctiluca* totivirus-like virus 1 (LnoTLV1) genome is 4761 nt long and contains two ORFs coding for proteins of 834 and 693 aa. As seen in Blastp search, the longer ORF was similar to hypothetical protein 3 of Hubei toti-like virus 16 (90% coverage with 24% identity, 1,923,304). HHpred found the longer ORF similar to RdRP of foot-and-mouth disease virus (*e*-value = 3.6e-24, 4WYW_A). According to Blastp search, the shorter ORF was similar to hypothetical protein 2 of Hubei toti-like virus 16 (99% coverage and 31% identity), whereas HHPred found no similar protein sequences. Hubei toti-like virus 16 is isolated form spiders [16]. According to phylogenetic analysis, LnoTLV1 was most similar to Hubei toti-like virus 16 and Beihai sea slater virus 3, isolated from wharf roach (1,922,659) (Online Resource 11).

### *Lampyris noctiluca* errantivirus 1

Errantiviruses are endogenous retroviruses of insects. *L. noctiluca* errantivirus 1 (LnoELV1) genome is 6948 nt long, with three ORFs in two reading frames and long-terminal repeats of 90 nucleotides. The largest ORF of 1096 aa is similar to uncharacterised protein of psyllid *Diaphorina citri* (83% coverage and 66% identity, 121,845) and ORF B of errantivirus Trichoplusia ni TED virus (81% coverage and 66% identity, 2,083,181) according to Blastp search. HHpred identified two sites similar to reverse transcriptase/ribonuclease H p80 of moloney murine leukemia virus (*e*-value = 2.5e-56, 4MH8_A) and to integrase of human spumaretrovirus (*e*-value = 1.6e-33, 3OYM_B). The 508 aa protein sequence was similar to several predicted insect proteins (such as *Tribolium castaneum*: XP_015840241.1, *Halyomorpha halys*: XP_024219554.1, and *Papilio machaon*: XP_014361342.1) and envelope protein of *Drosophila melanogaster* (79% coverage and 27% identity, CAA04048.1), according to Blastp search. HHpred found protein similarity to fusion protein of newcastle disease virus (*e*-value = 0.0098, 1G5G_C). Blastp search found the shortest ORF of 317 aa to be similar to ORF A of Trichoplusia ni TED virus (70% coverage and 33% identity). According to ICTV, species of errantiviruses have generally less than 50% identity in their Gag protein. HHpred showed that the protein was similar to gag protein of rous sarcoma virus (*e*-value = 5.6e-13, 5A9E_B). In phylogenetic analysis, LnoErV1 was found to be similar to uncharacterized proteins from several insects, such as, kissing bug, the old world swallowtail butterfly, fruit fly, louse, termite, ant, and silkworm, and transposons from the cabbage looper moth and red flour beetle, indicating that these insects have similar endogenous retroviruses (Online Resource 11).

### Glow-worm viruses in other insects

We used NCBI’s transcriptome shotgun assembly sequence database (TSA) to search for similar virus sequences in other insects. We set Blastx bit score, which takes into account the alignment length, mismatches and gaps, above 200 for positive hits. We found no similar viruses from insect transcriptomes for LnoFV1, LnoPLV2, or LnoTLV1 (Table 1). However, for the other viruses, we found similar sequences from several insect hosts, ranging from fruit flies and mosquitoes to bugs and sharpshooters (Table 1 and Online Resource 5–12). Interestingly, we found that a firefly (*Photinus pyralis*) shared five similar virus sequences (LnoIV1, LnoIV2, LnoBLV1, LnoPLV1, and LnoErV1) with glow-worms. The western tarnished plant bug (*Lygus hesperus*) shared four similar virus sequences (LnoIV1, LnoIV2, LnoBLV1, and LnoErV1), and the mediterranean fruit fly (*Ceratitis capitata*) and sharpshooter (*Cuerna arida)* shared three similar viruses (LnoIV1, LnoIV2, and LnoRLV1). All the insect hosts that had LnoIV1-like virus sequences also had LnoIV2-like virus.

### The glow-worm virus loads, prevalence, and interactions

**Fig. 2:**
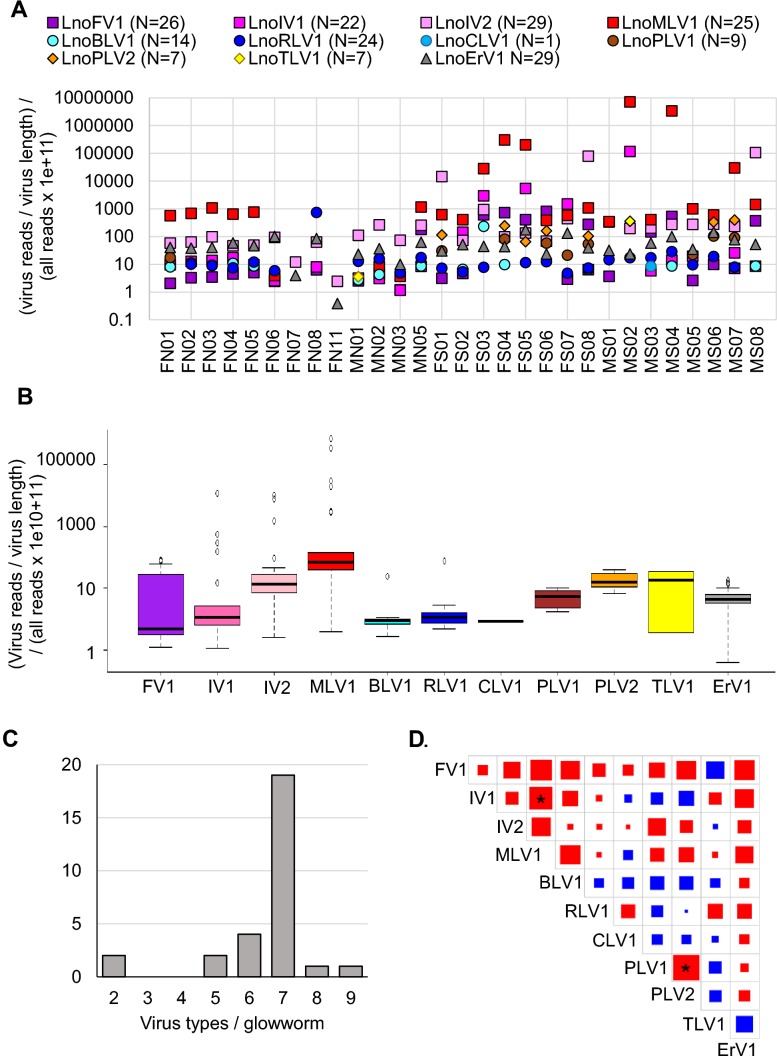
Glow-worm RNA virus loads, prevalence, and interactions: **a** individual standardized read amounts for the 11 viruses were plotted for each glow-worm (*N* = 29). Virus color code is given above the scatter plot. Females and males from the northern population (Konnevesi) are marked FN and MN, and females and males from the southern population (Hanko) are marked FS and MS, respectively. **b** Each glow-worm virus has its typical load levels. Box plots show variation of virus-specific reads in the glow-worms. Black line represents median value, values inside the box cover first and third quartile (interquartile) range, the whisker show values up to 1.5 times the interquartile range, and gray circles are individual values beyond the whisker range (outliers). **c** Glow-worms infected, on average, by seven different virus types. **d** Possible virus interactions were analyzed by pairwise comparison of virus read amounts with Spearman’s correlation coefficient. Positive correlation is indicated by red, negative correlation by blue, and the magnitude of the adjusted p-values is indicated by the size of the colored square. The two statistically significant interactions are shown by asterisk: LnoIV1 and LnoMLV1, and LnoPLV1 and LnoPLV2 virus loads correlated positively with each other

Viral RNA read amounts for samples collected in 2017 were analyzed for each glow-worm individually. Before the analysis, we removed poly-A-tails from the virus sequences. Only reads with a properly paired mate read mapping to the virus sequence were analyzed and the results were standardized by virus length and all clean reads of the sample (Fig. 2a). The most common viruses were LnoIV2 and LnoErV1, which were found in all samples. A high prevalence of LnoErV1 is expected as it is probably integrated into the glow-worm genome. The least common virus, found only in one sample, was LnoCLV1. The two Finnish glow-worm populations, southern Hanko and northern Konnevesi, had significantly different amounts of LnoFV1 (two-tailed student’s *t* test: *t* = − 3.14, *p*-value = 0.0068 for females, not significant in males), LnoPLV1 (*t* = − 2.73, *p*-value = 0.015 for females, not significant in males), and LnoPLV2 (*t* = − 2.94, *p*-value 0.01 for females, not significant in males). LnoFV1 load levels were higher in Hanko than those in Konnevesi population and partiti-like viruses were almost exclusively found in the Hanko population.

Viral load variation was analyzed for each virus separately (Fig. 2b). The positive-strand RNA virus loads varied somewhat more than the other RNA viruses; for example, positive-strand RNA viruses had a larger interquartile value range and more outlier values. Especially, LnoMLV1 had five outlier values, of which the highest reached over 1000 times higher than the median value. Two of the highest LnoMLV1 amounts were found in the two southern population male heads, 48% and 23% of all reads were coding for LnoMLV1. The highest virus read amounts in female lanterns coding for LnoMLV1 were 2% and 1.4% of all reads. There were no differences in the total viral load between the Hanko and Konnevesi populations (*t* = 0.08, *p*-value = 0.94).

Glow-worms, irrespective of their gender or population, had seven different virus types, on average. A minimum of two and at the most nine virus types were observed (Fig. 2c). We studied whether the viruses affected each other’s infection by pairwise correlations (Fig. 2d) and found two statistically significant interactions, read counts of LnoIV1 and TyLV1 correlated positively (Spearman’s correlation coefficient = 0.6, Holm’s method adjusted *p*-value = 0.036) and existence of the two partiti-like viruses correlated positively in the individuals (correlation coefficient = 0.8, adjusted *p*-value < 0.0001).

### Glow-worm virus loads in different body sections

We studied the virus loads by qPCR of glow-worm samples collected in 2018. Four female samples from Äänekoski and five female samples from Tvärminne were separated into head (the first segment, H), body (B), and eggs (E). The females were most probably unmated since they were still glowing when they were collected. Egg samples were scraped from body cavity and may have contained other tissues such as fat [29].

**Fig. 3:**
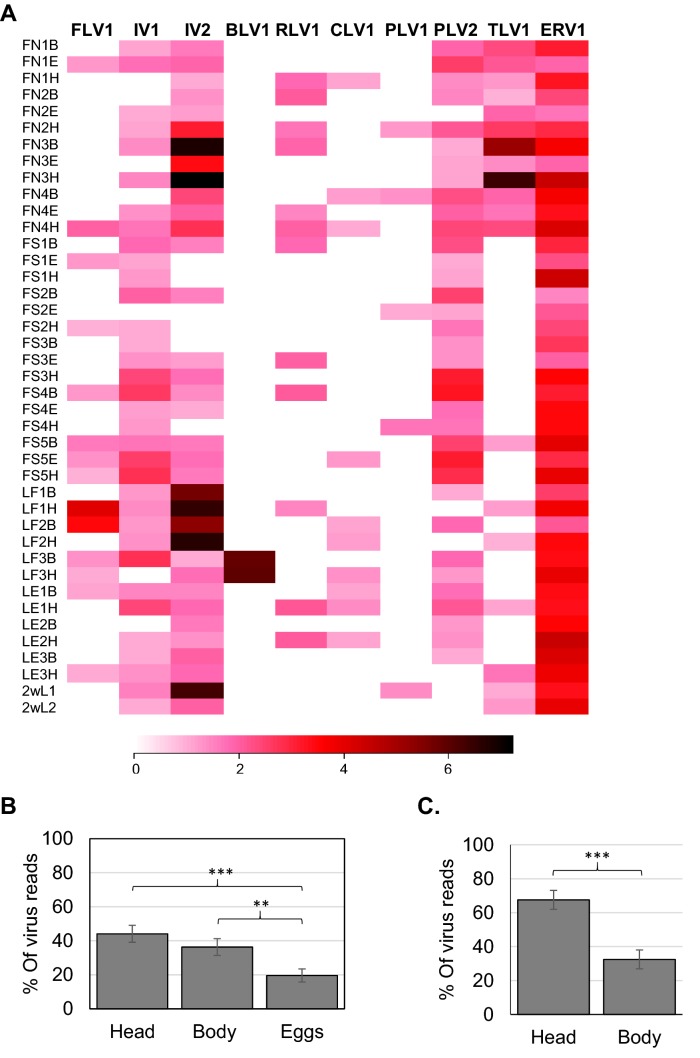
Virus loads in 2018 glow-worm samples: **a** the glow-worm RNA virus levels were studied by qPCR from head (H), body (B), and eggs (E) of nine Finnish glow-worm females from two populations (FN: Äänekoski and FS: Tvärminne) and from heads (H) and bodies (B) of three laboratory-reared larvae from Finland (LF) and three from England (LE). Additionally, 12 unfed 2-week-old larvae split into two pools (2wL1 and 2wL2) were also studied. Virus loads in 10-based logarithmic scale shown in a heat map from white (no virus) to dark red (loads over 1,000,000 virus sequences per sample). The bar blots show the virus distribution between the different body parts in **b** females (*N* = 7) and **c** Larvae (*N* = 6). The sum of virus reads in all of the body parts is 100%. Variation is shown by standard error and significant differences between groups by asterisk (*** = *p*-value < 0.001, and ** = *p*-value < 0.01)

Additionally, three laboratory-reared larvae from southern Finland (Hanko) and three from southern England (Princes Risborough) were divided into head (H) and body (B) sections. Twelve isolated and unfed 2-week-old larvae from a single southern Finnish mother were split into two groups with six larvae in each (2wL1 and 2wL2) to yield enough RNA for experiment. All the viruses characterized from the 2017 samples, except LnoMLV1, were found from the 2018 female samples and their eggs (Fig. 3a), indicating that the viruses may be vertically and maternally transmitted. In further support of vertical transmission, we found LnoIV1, LnoIV2, LnoPLV1, LnoTLV1, and LnoERV1 from the unfed larval samples. Furthermore, all the viruses except LnoPLV1 and LnoMLV1 were also in the laboratory-reared larvae. Similar to the RNA-seq data, the prevalence of LnoRLV1 (67%), LnoFV1 (73%), LnoIV1 (100%), LnoIV2 (100%), and LnoERV1 (100%) were high. Yet the prevalence of LnoPLV2, which was 24% in the 2017 data and was found to be 100% in the 2018 data. Further differences in LnoBLV1virus prevalence between the sample cohorts were 48% in the 2017 samples and 7% in 2018 samples, and for LnoCLV1, the prevalence was 3% in 2017 samples and 50% in 2018 samples.

In females, there was no significant difference in the distribution of viruses between head and body (*N* = 55, *t* = − 1.12, *p*-value = 0.27). However, the egg tissue had significantly lesser viruses than those in heads (*t* = − 3.94, *p*-value = 0.00014) or bodies (*t* = − 2.695, *p*-value = 0.0081, Fig. 3b). In larvae, the heads had a significantly higher percentage of viruses than those in the bodies (*N* = 36, *t* = 4.45 *p*-value = 0.00003, Fig. 3c).

There were no differences between populations in the virus load (mean + SE: Tvärminne 12,876 + 4581 and Äänekoski 9132.80 + 4874, t = 0.55, p-value = 0.60). We excluded FN3 sample (Fig. 3a) from the analyses, as it had very high virus load.

## Discussion

We identified 11 novel common glow-worm RNA viruses. In insects, viruses transmit horizontally through feeding, mating, or a vector [30]. Vertical virus transmission may occur through genome integration, such as for retroviruses or wasp polydnaviruses [31], or infection of eggs and sperm. Strict vertical transmission by infection of eggs or sperm is documented only for Drosophila sigmavirus [32]. Several insect viruses studied in bees and mosquitoes can transmit both horizontally and vertically [33, 34]. Vertical transmission has been hypothesized to be associated with low virulence and latent infection while under certain conditions, such as during host stress or presence of secondary hosts, virus activation enables horizontal transmission [33, 34]. Since glow-worms are in contact with each other only at birth, when foraging during larval stage, and during mating, within population transmission of viruses would be inefficient without vertical transmission.

However, horizontal transmission to and from mollusc is possible. Larvae had most of the viruses in their head segment, which may indicate possibility of horizontal transmission as the larvae are predators and salivary glands are known sites to carry viruses [35]. Adults do not feed and have no mouthparts and, consistently, had no significant differences in virus loads between head and body. Glow-worm adults and larvae are unlikely to be eaten by other insects as larvae and probably also adults are distasteful [1, 3] and thus this kind of virus transmission pathway is not expected.

Most of the identified viruses were found from the glow-worms collected both in 2017 and in 2018 suggesting that they form stable infections. As we found the same RNA virus sequences from the four spatially separate Finnish populations, we suspect that these viruses are a natural set of viruses infecting glow-worms and not an indication of a pathogenic state. However, LnoTyLVI is an exception. We could not find LnoMLV1 from the 2018 samples, and LnoMLV1 loads were extremely high in some of the 2017 samples: in the two male heads their amounts reached up to 48% and 23% of all the sequenced RNA reads. In comparison to negative- and double-stranded viruses, RNA levels of positive-strand RNA viruses, such as LnoMLV1, varied most between the glow-worm individuals. Similar phenomenon has also been seen in Argentine ant viruses [36]. However, such massive LnoMLV1 levels as found in the two males are clearly a result of pathogenic virus activation and might explain why we did not detect LnoMLV1 the next year. Viruses can be very reactive in a new host but then disappear suddenly if they fail to adjust and transmit [37].

Interestingly, we found five similar viruses from RNA-sequencing data of another glowing insect: the common eastern firefly (*Photinus pyralis*). The common glow-worm and the eastern firefly are from the same Lampyridae family, which may explain why they share similar viruses. Yet, they do not occur in the same continent: eastern firefly is found in North America and common glow-worm only in Eurasia [4]. However, we did not find from our data the two orthomyxo-like viruses, which were identified from the common eastern firefly and shown to transmit vertically [8].

As the light emitting ability of glow-worms is a major fitness factor, it will be interesting to study whether viruses affect the bioluminescence in larvae or females. According to our study, a whole range or viruses reside at lantern tissue so the light-regulating interaction could be possible.

## Electronic supplementary material

Below is the link to the electronic supplementary material.

Supplementary file1 (DOCX 153 kb)

Supplementary file2 (DOCX 12 kb)

Supplementary file3 (DOCX 1705 kb)

Supplementary file4 (DOCX 13 kb)

Supplementary file5 (PDF 23 kb)

Supplementary file6 (PDF 64 kb)

Supplementary file7 (PDF 34 kb)

Supplementary file8 (PDF 40 kb)

Supplementary file9 (PDF 41 kb)

Supplementary file10 (PDF 38 kb)

Supplementary file11 (PDF 31 kb)

Supplementary file12 (PDF 27 kb)

## Data Availability

The datasets generated and analyzed during this study are available with the corresponding author, on reasonable request.
