# Supplementary figures and images for: Identification and characterisation of common glow-worm RNA viruses
Source: Virus Genes. 2020 Jan 3;56(2):236–48. doi: 10.1007/s11262-019-01724-5 (PMC7093385; doi:10.1007/s11262-019-01724-5)

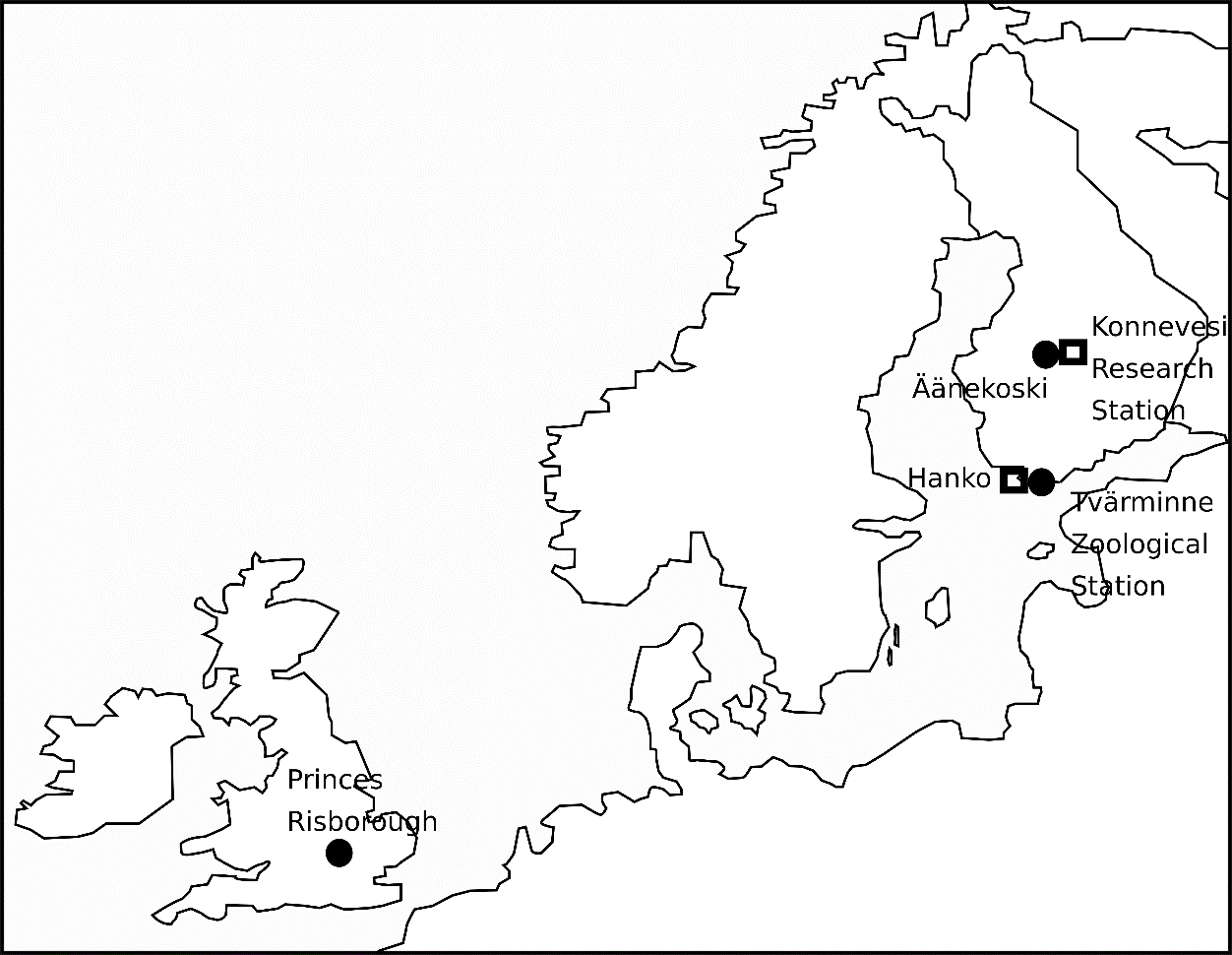

Supplement: Supplementary file 1 — Supplementary file1 (DOCX 153 kb) [file 11262_2019_1724_MOESM1_ESM.docx]

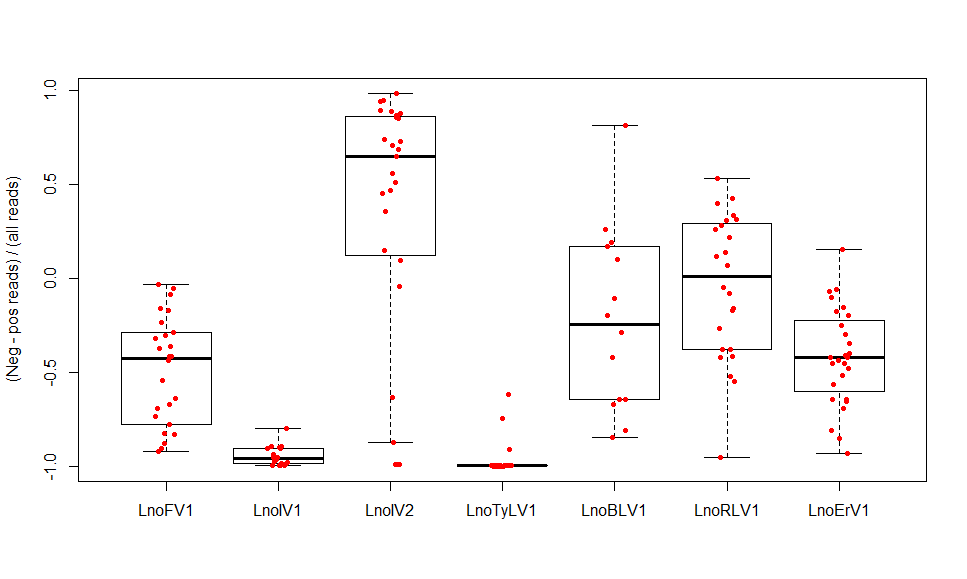


**LnoFV1 LnoIV1 LnoIV2 LnoMLV1 LnoBLV1 LnoRLV1 LnoErV1**

Supplement: Supplementary file 3 — Supplementary file3 (DOCX 1705 kb) [file 11262_2019_1724_MOESM3_ESM.docx]

Tree scale: 0.1

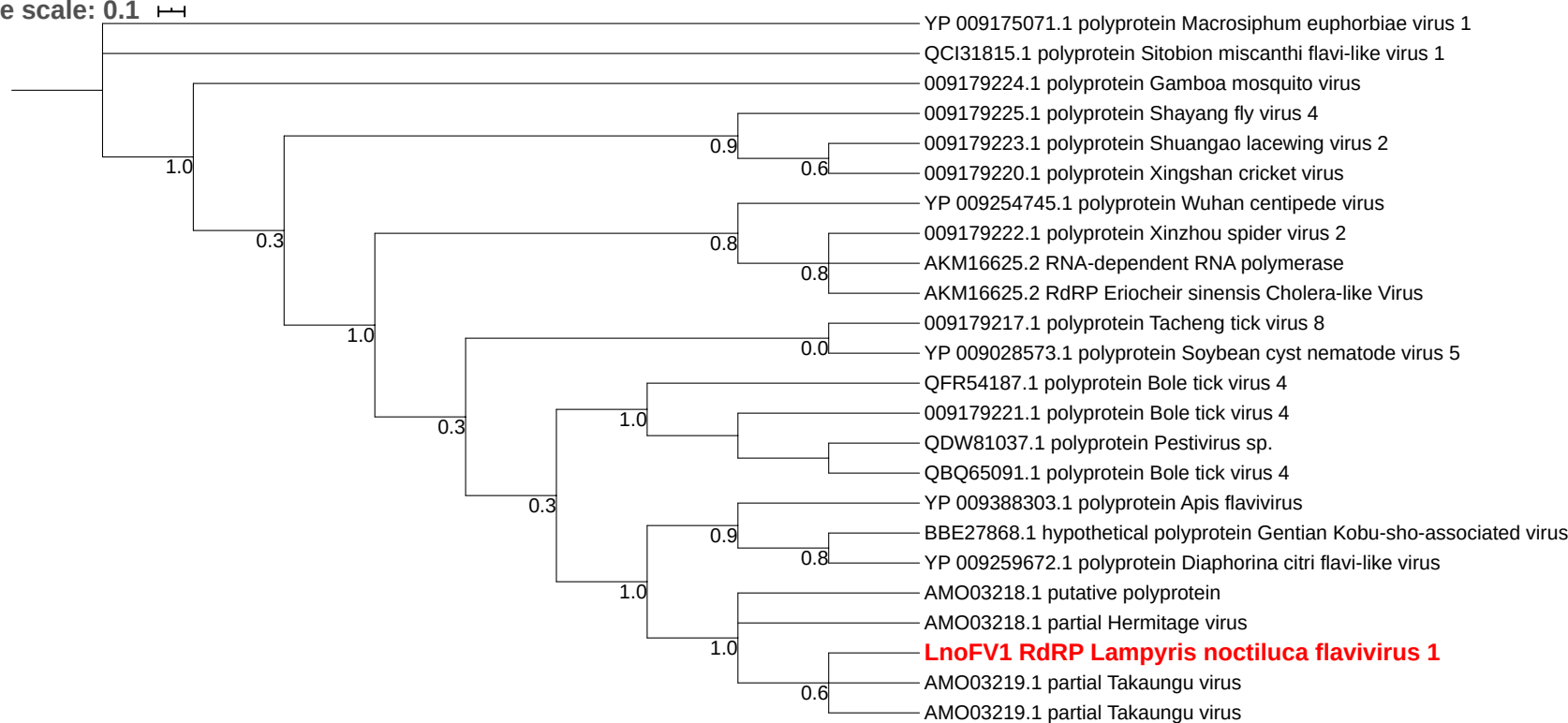

Supplement: Supplementary file 5 — Supplementary file5 (PDF 23 kb) [file 11262_2019_1724_MOESM5_ESM.pdf]

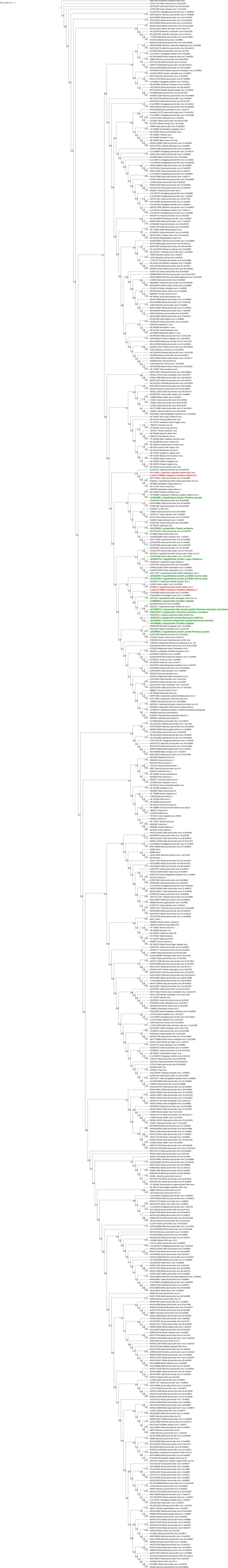

Supplement: Supplementary file 6 — Supplementary file6 (PDF 64 kb) [file 11262_2019_1724_MOESM6_ESM.pdf]

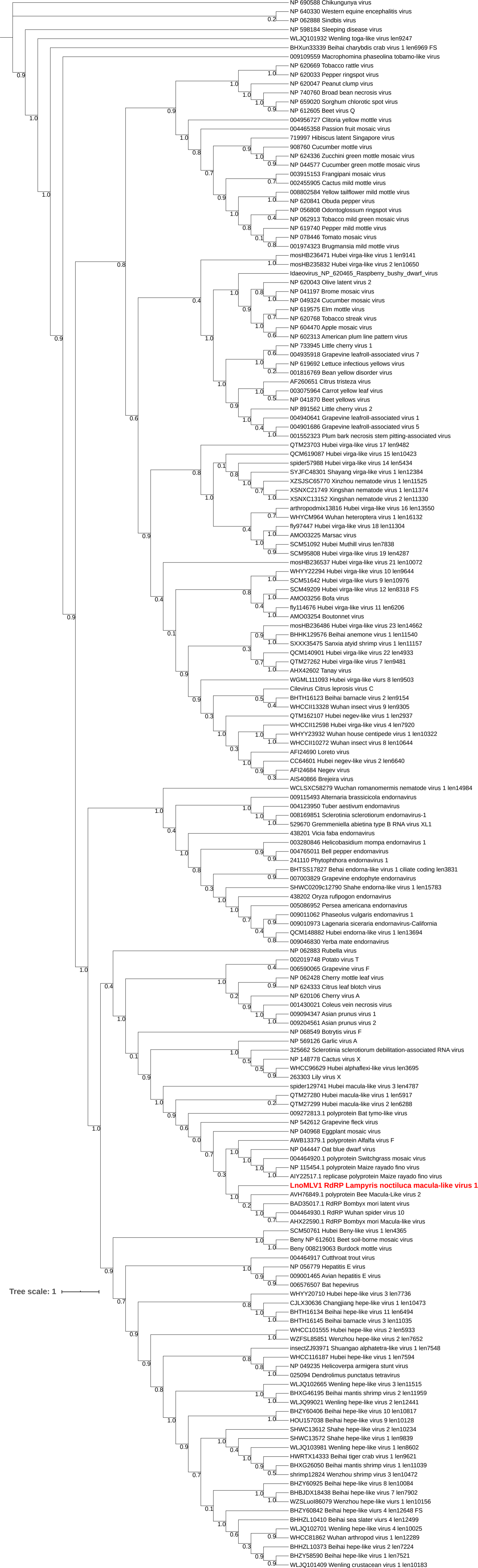

Supplement: Supplementary file 7 — Supplementary file7 (PDF 34 kb) [file 11262_2019_1724_MOESM7_ESM.pdf]

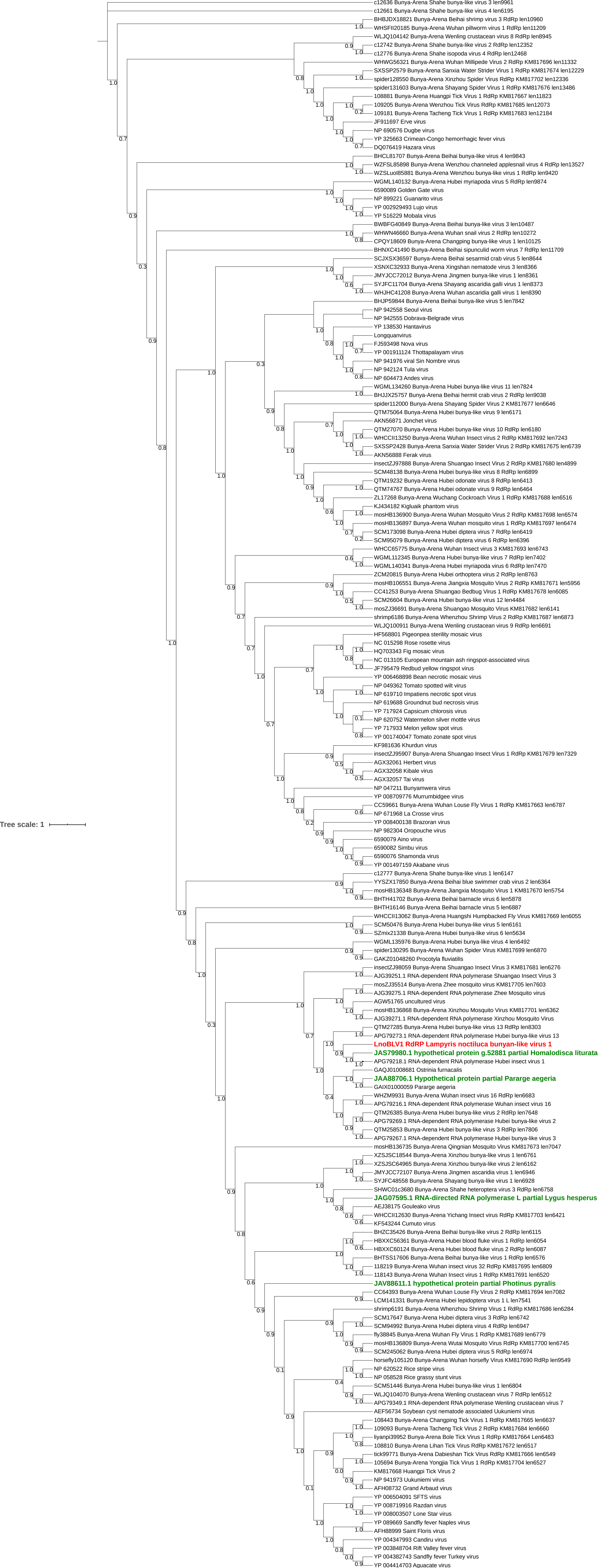

Supplement: Supplementary file 8 — Supplementary file8 (PDF 40 kb) [file 11262_2019_1724_MOESM8_ESM.pdf]

Tree scale: 1

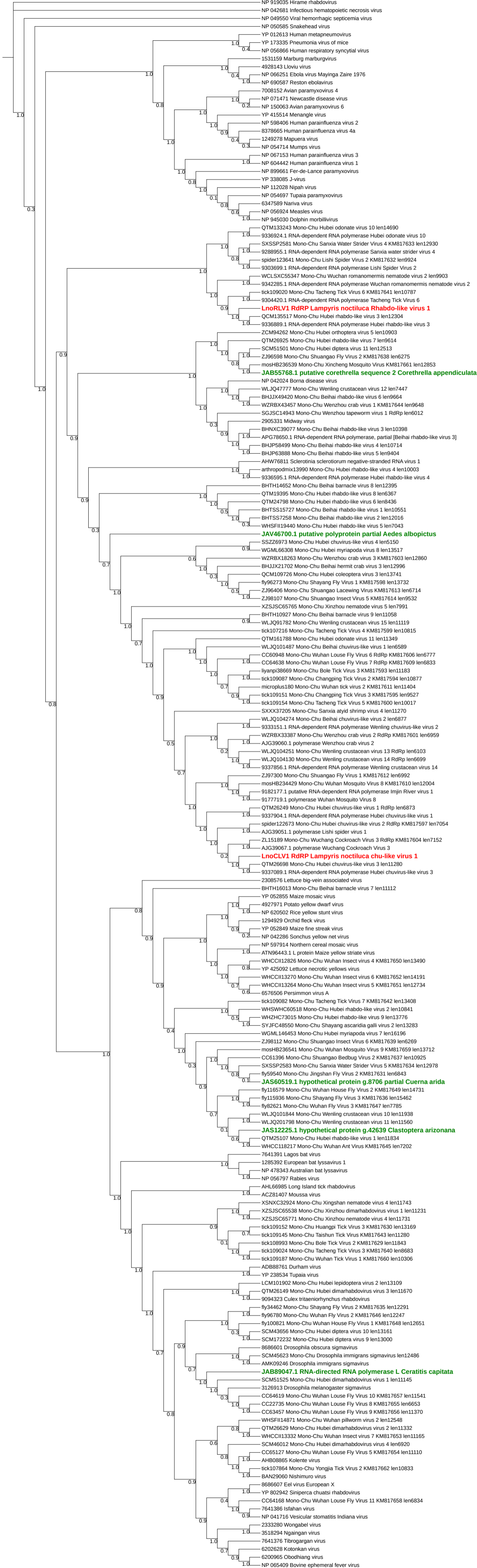

Supplement: Supplementary file 9 — Supplementary file9 (PDF 41 kb) [file 11262_2019_1724_MOESM9_ESM.pdf]

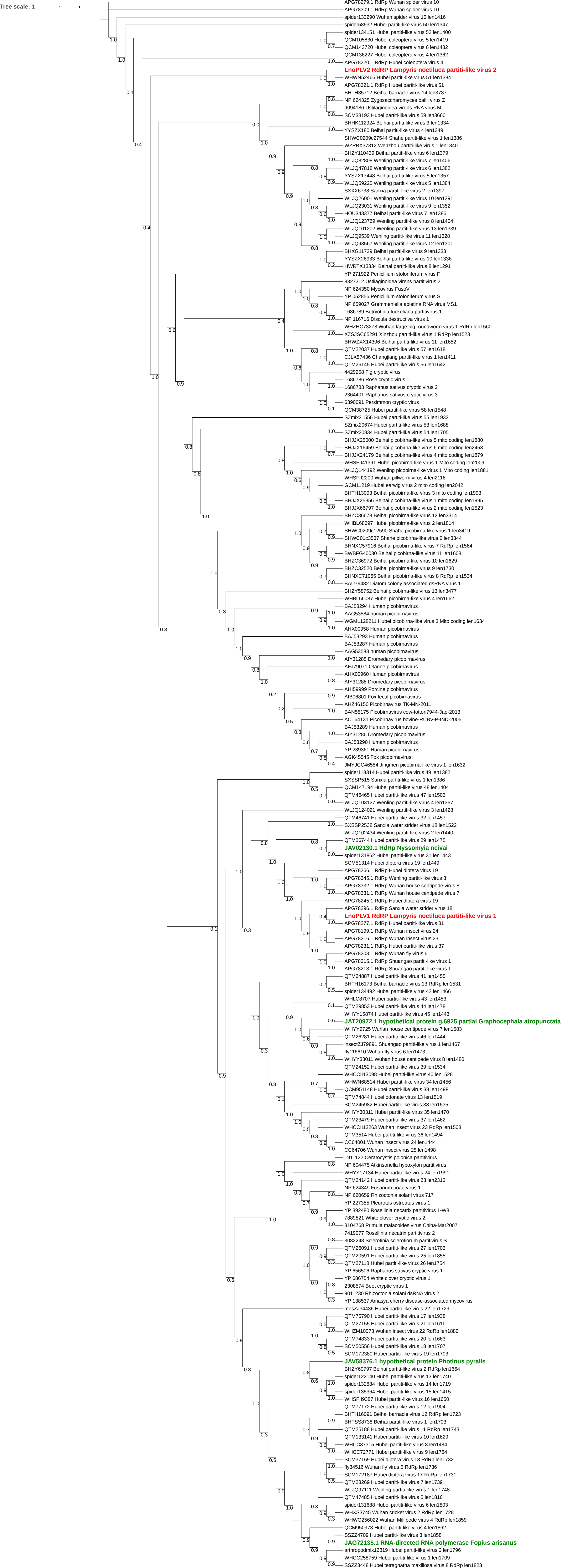

Supplement: Supplementary file 10 — Supplementary file10 (PDF 38 kb) [file 11262_2019_1724_MOESM10_ESM.pdf]

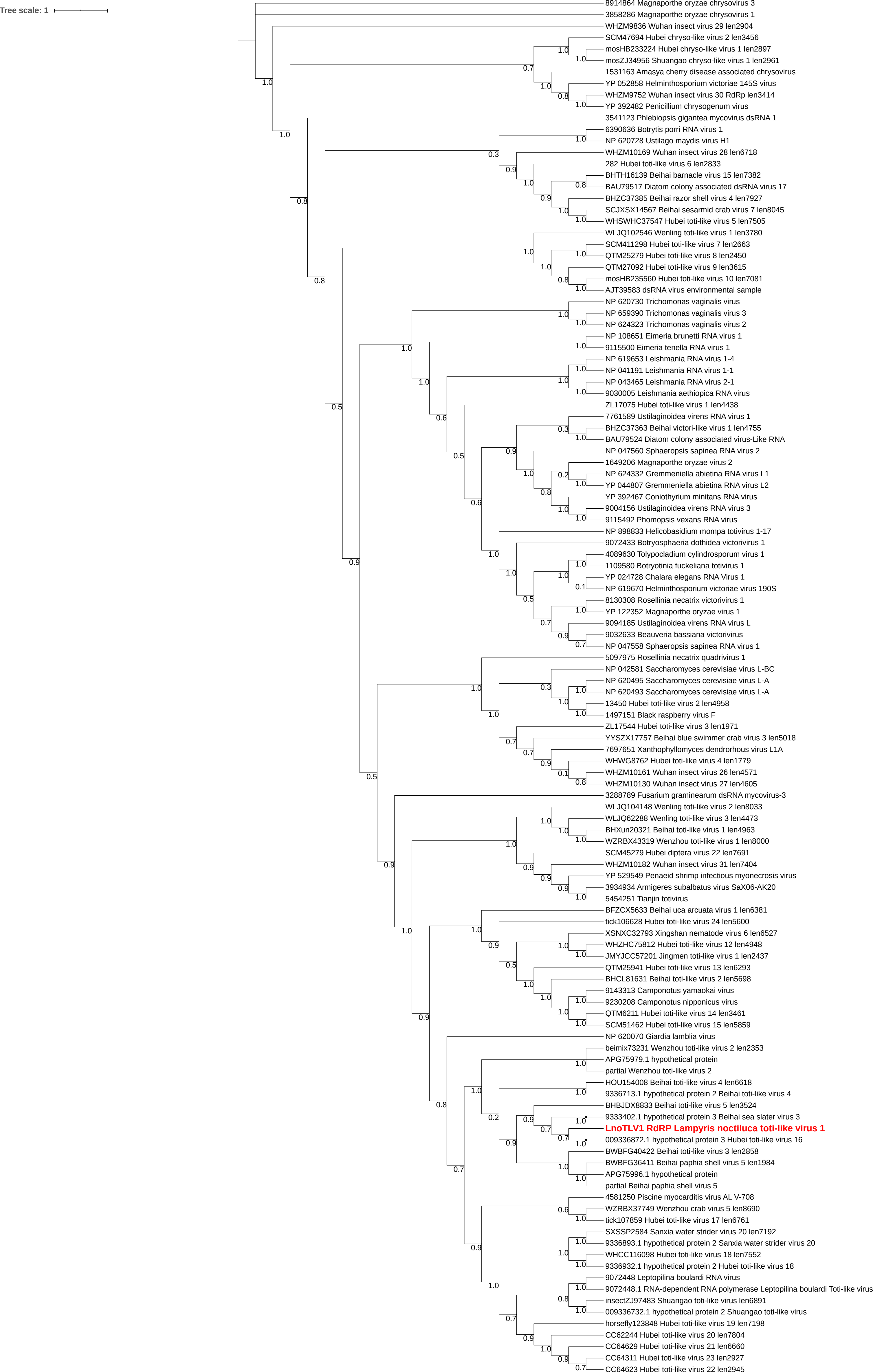

Supplement: Supplementary file 11 — Supplementary file11 (PDF 31 kb) [file 11262_2019_1724_MOESM11_ESM.pdf]

Tree scale: 0.1

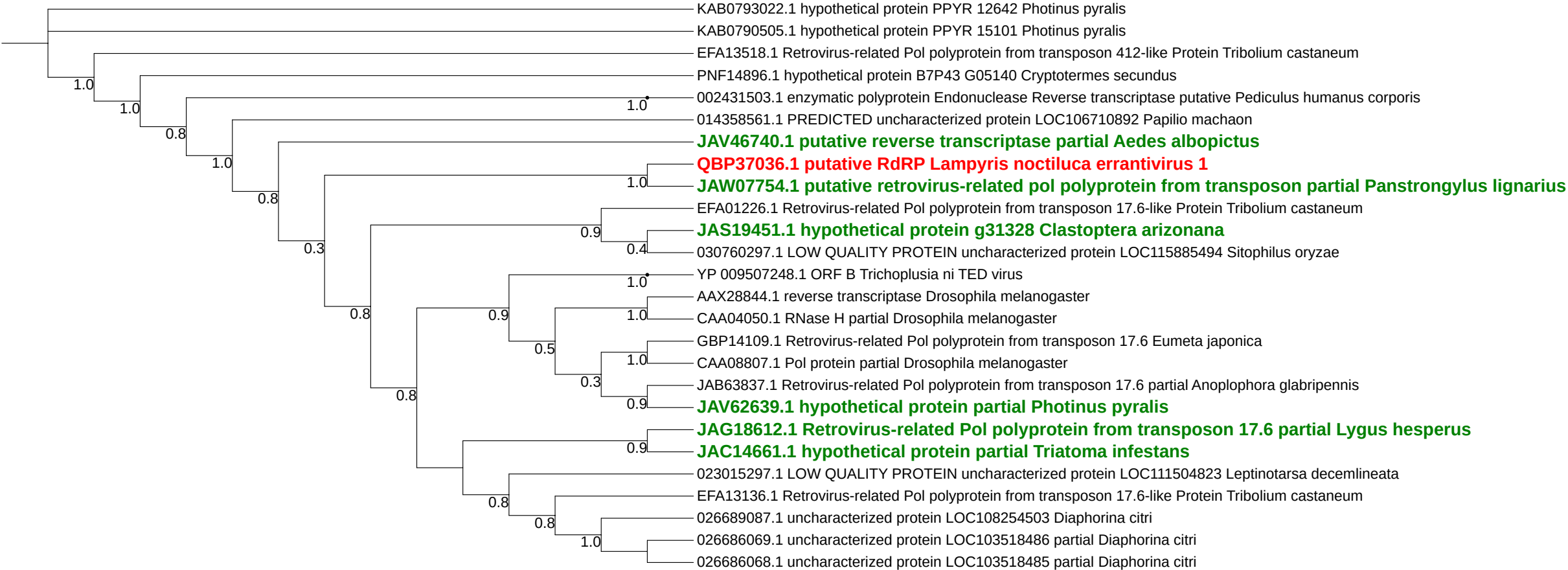

Supplement: Supplementary file 12 — Supplementary file12 (PDF 27 kb) [file 11262_2019_1724_MOESM12_ESM.pdf]
