# Supplementary material for: Identification and characterisation of common glow-worm RNA viruses
Source: Virus Genes. 2020 Jan 3;56(2):236–48. doi: 10.1007/s11262-019-01724-5 (PMC7093385; doi:10.1007/s11262-019-01724-5)
Supplement: Supplementary file 2 — Supplementary file2 (DOCX 12 kb) [file 11262_2019_1724_MOESM2_ESM.docx]

| Virus | Forward primer 5’ | Reverse primer 5’ |
| --- | --- | --- |
| LnoFV1 | TTGAATGAAACTGCGGTGGA | ACCTTGGTATGCAAAAGCCA |
| LnoIV1 | ATTGACCCCGCTACGTTTAC | CAAGCTTTTCACATCGCCTG |
| LnoIV2 | ATTTCCCTGATCCGCAACGT | TCCGGCAACATATCCCCAAC |
| LnoMLV1 | GATTTCTTCTGCCGTCACGC | CGAGAAAGCCAAGCACAACC |
| LnoBLV1 | GGACTGCTGGGGTTGTCTTT | ACCATCATCTCCCACTTCCA |
| LnoErV1 | TGCACCAAGTACATTCCAAAGA | ATGTTCTTGCAGTGATGTTCCT |
| LnoRLV1 | TGGGAGCCAATCATTCAGGG | TATGTCCGGGCTTGCATCTG, |
| LnoCLV1 | ACTATTGGGACGGACAAGCC | GGTATTTCAGAAGCAGCGCG |
| LnoPLV1 | CGACCCGTATGCATTCCAGA | TCACCCCGTTCGACAAGATG |
| LnoPLV2 | GCCCACTAAGACCTATGCCC | GCATCTCAAAATCGGGTGGC |
| LnoTLV1 | CCTGGACCCATGAAAGCTGT | GCTTATAGTCCGGCCCTCAC |
