# Supplementary material for: Identification and characterisation of common glow-worm RNA viruses
Source: Virus Genes. 2020 Jan 3;56(2):236–48. doi: 10.1007/s11262-019-01724-5 (PMC7093385; doi:10.1007/s11262-019-01724-5)
Supplement: Supplementary file 4 — Supplementary file4 (DOCX 13 kb) [file 11262_2019_1724_MOESM4_ESM.docx]

| **LnoMLV1** | **N** | **No. of variable sites** | **No. Of variable sites / genome size / N** |
| --- | --- | --- | --- |
| North females | 5 | 54 | 0.0016 |
| North males | 1 | NA | NA |
| North all | 6 | 283 | 0.0070 |
| South females | 8 | 436 | 0.0081 |
| South males | 7 | 439 | 0.0093 |
| South all | 15 | 523 | 0.0052 |
| All | 21 | 527 | 0.0037 |
| **LnoFV1** |  |  |  |
| North females | 0 | NA | NA |
| North males | 1 | NA | NA |
| North all | 1 | NA | NA |
| South females | 5 | 90 | 0.00097 |
| South males | 1 | NA | NA |
| South all | 6 | 222 | 0.0020 |
| All | 7 | 687 | 0.0053 |
| **LnoIV1** |  |  |  |
| South females | 3 | 301 | 0.0097 |
| South males | 0 | NA | NA |
| South all | 3 | NA | NA |
| All | 3 | NA | NA |
| **LnoIV2** |  |  |  |
| South females | 4 | 636 | 0.015 |
| South males | 1 | NA | NA |
| South all | 5 | 638 | 0.012 |
| All | 5 | NA | NA |
| **LnoPLV1** |  |  |  |
| South females | 2 | 3 | 0.0010 |
| South males | 3 | 5 | 0.0011 |
| South all | 5 | 9 | 0.0012 |
| All | 5 | NA | NA |
| **LnoPLV2** |  |  |  |
| South females | 3 | 7 | 0.0016 |
| South males | 4 | 8 | 0.0014 |
| South all | 7 | 15 | 0.0015 |
| All | 7 | NA | NA |
